# Empowering Indigenous Health: A Call for Equity and Innovation in Public Health

**DOI:** 10.3389/ijph.2025.1607763

**Published:** 2025-06-20

**Authors:** Nurul Athirah Naserrudin, Pauline Yong Pau Lin

**Affiliations:** ^1^ Institute for Health Systems Research, National Institutes of Health, Setia Alam, Malaysia; ^2^ Sociology and Social Anthropology Program, Faculty of Social Sciences and Humanities, Universiti Malaysia Sabah, Kota Kinabalu, Sabah, Malaysia

**Keywords:** indigenous healthcare, equity, community based healthcare, citizen science, rural healthcare

**The IJPH series “Young Researcher Editorial” is a training project of the Swiss School of Public Health.**

The WHO’s Sustainable Development Goals and the Global Roadmap for Healthy Longevity emphasise diversity, equity, and inclusivity (DEI) in public health strategies [1]. Governments must offer tailored, sustainable solutions to health inequities while building resilience [2]. For indigenous populations, which disproportionately face health inequity, DEI depends on integrating participatory approaches, traditional knowledge, and equitable policies into public health initiatives, centering the voices and experiences of indigenous communities.

In participatory approaches such as Community-Based Participatory Research (CBPR) and citizen science, indigenous populations are actively involved in shaping and implementing health interventions. CBPR focuses communities on the issues they identify and promotes shared decision-making and co-creation throughout the research process [2]. In citizen science, researchers often define questions, and community members collect and help analyse data, creating opportunities for ownership, collaboration, and inter-generational knowledge exchange [3]. Indigenous participation ensures that health initiatives are inclusive, culturally relevant, meet community needs, and align with community values and priorities. For example, in Canada, the Outdoor Adventure Leadership Experience (OALE) program was co-developed with the Wikwemikong First Nation to promote adolescent wellbeing through culturally grounded land-based leadership training, resulting in a community-owned and sustained intervention [3]. Similarly, in the United States, long-term CBPR partnerships coordinated by the Centre for Native Health Partnerships demonstrated how trust, cultural humility, and shared governance are critical for addressing health disparities across tribal nations​ [4].

Incorporating traditional knowledge into public health strategies can enhance cultural relevance, community trust and effectiveness. Indigenous elders, healers, and midwives, hold a wealth of knowledge about culturally grounded practices, kinship systems, and intergenerational caregiving that are important to community wellbeing [5] Systematic reviews have highlighted the potential for such knowledge systems to improve health outcomes when integrated through respectful, reciprocal, and community-led processes [6]. Traditional midwifery has improved safe childbirth practices in many indigenous settings, demonstrating how culturally tailored practices can complement modern healthcare systems [5]. Structured mechanisms, such as advisory platforms that include indigenous representatives, capacity-building programs for health professionals and policymakers, and equitable resource allocation, can facilitate the integration of traditional knowledge into formal health systems [6].

Despite their potential, these approaches face barriers. Policymakers often lack an understanding of the cultural practices central to indigenous health, and the process of standardising diverse traditional practices remains a complex challenge [7]. Overcoming these barriers requires consistent dialogue between indigenous communities and stakeholders, developing collaboration through mutual respect and shared goals. Collaborative platforms that document and disseminate traditional practices can help policymakers appreciate their value, enhancing their visibility and utility in health systems, while piloting programs for the evaluation and refinement of culturally appropriate interventions before scaling nationally [7].

Improving community health infrastructure plays an important role in tackling health inequalities. Services such as mobile clinics, telehealth, access to clean water, and financial support have shown positive outcomes in reaching underserved populations [3, 4, 6]. For ageing Indigenous communities, especially during health emergencies like the COVID-19 pandemic, healthcare must be shaped by local knowledge and cultural practices. In countries like the United States, Canada, and Australia, indigenous groups experienced greater risks due to long-standing structural barriers and a lack of culturally safe services. Despite these challenges, many communities took action through their own systems of care, highlighting the value of indigenous leadership and decision-making [8]. Building trust, ensuring cultural safety, and involving local leaders have been shown to support better ageing experiences. Creating person-centred, community-based health systems that draw on indigenous knowledge and involve local health workers is one way to reduce ongoing service gaps [9, 10]. These examples point to the importance of healthcare models that are culturally relevant, fair, and led by the communities they aim to serve.

The evidence highlights that achieving healthy longevity for indigenous populations requires a shift toward holistic models of care that incorporate participatory methods, respect traditional knowledge and are guided by equity-focused policies. These strategies address immediate health needs and contribute to building resilient health systems capable of supporting indigenous communities through future challenges. By prioritising collaboration, equitable resource allocation, and culturally sensitive practices, public health professionals and policymakers can create inclusive health systems that support healthier, more fulfilling lives for indigenous populations as they age. Sustained commitment to these approaches will ensure that public health initiatives are effective, equitable, and reflective of the unique needs of indigenous communities.

## Data Availability

The original contributions presented in the study are included in the article/supplementary material, further inquiries can be directed to the corresponding author.
